# Blocking Two-Pore Domain Potassium Channel TREK-1 Inhibits the Activation of A1-Like Reactive Astrocyte Through the NF-κB Signaling Pathway in a Rat Model of Major Depressive Disorder

**DOI:** 10.1007/s11064-023-03857-4

**Published:** 2023-01-20

**Authors:** Ting Cong, Ye Sun, Yitong Zhou, Haikuo Wu, Liya Li, Zhenchen Chu, Xue Chen, Jinying Li, Danmei Zhao, Yanfang Wang, Yingxin Liu, Shengming Yin, Zhaoyang Xiao

**Affiliations:** 1 Department of Anesthesiology, The Second Affiliated Hospital of Dalian Medical University, 467 Zhongshan Road, Shahekou District, Dalian, 116027 Liaoning China; 2 Department of Orthopedics, The First Affiliated Hospital of Dalian Medical University, Dalian, 116011 Liaoning China; 3 Department of Physiology, Basic Medicine College of Dalian Medical University, No. 9, West Section, Lvshun South Road, Lvshunkou District, Dalian, 116044 Liaoning China

**Keywords:** Major depressive disorder, TREK-1, A1-like reactive astrocyte, CUMS, NF-κB signaling pathway

## Abstract

Major depressive disorder (MDD) refers to a widespread psychiatric disorder. Astrocytes play a pivotal role in regulating inflammation which is a well-acknowledged key component in depression pathogenesis. However, the effects of the neuroinflammation-inducing A1-like astrocytes on MDD are still unknown. TWIK-related K^+^ channel 1 (TREK-1) has been demonstrated to regulate the action of antidepressants. Nevertheless, its mechanisms and effects on A1-like astrocyte stimulation in MDD are not clear. Therefore, we conducted in vivo and in vitro experiments using TREK-1 specific inhibitor spadin. In vivo, rats were subjected to a 6-week chronic unpredictable mild stress (CUMS) followed by spadin treatment. Behavioral tests were employed to surveil depressive-like behaviors. Hippocampal proteomic analysis was carried out with the purpose of identifying differentially expressed proteins after CUMS and spadin treatments. In vitro, astrocyte-conditioned medium and spadin were used to treat rat astrocyte cell line. The activated microglia, inflammatory factors, A1 astrocyte markers, and activated nuclear factor kappa B (NF-κB) pathway were later analyzed using immunofluorescence, western blot, and RT-qPCR. Our findings indicated that blockage of TREK-1 reduced CUMS-induced depressive-like behavior in rats, inhibited the microglial stimulation, reduced inflammatory factor levels, and suppressed the activation of A1-like reactive astrocytes in the hippocampus. We also verified that the suppression of A1-like astrocytes by spadin necessitated the NF-κB pathway. According to the findings, blocking TREK-1 inhibited the activation of A1-like reactive astrocytes via the NF-κB signaling pathway in MDD. Our study preliminarily identifies a novel antidepressant mechanism of TREK-1 action and provides a therapeutic path for MDD.

**Supplementary Information:**

The online version contains supplementary material available at 10.1007/s11064-023-03857-4.

## Introduction

Major depressive disorder (MDD) refers to a heterogeneous psychiatric condition with a global prevalence of over 300 million people [1] and constituting a major cause of disability worldwide [2]. Symptoms of MDD include relapsing episodes of desolation and despondency accompanied by anhedonia, concentration loss, low energy, alterations of memory, and relapsing suicidal intention, all linked to neurochemical and structural deficiencies [3, 4]. MDD is a multifactorial condition with varying etiological factors, such as genetic predisposition, a variety of stress, and other pathological events like inflammation [5]. However, its exact etiopathogenesis is insufficiently clarified so far.

As the most abundant cells of the central nervous system (CNS), astrocytes are involved in diverse functions like neuronal growth facilitation, synapse establishment, and blood brain barrier formation, as well as exert a pivotal inflammation-regulatory action [6]. Upon impairment of the brain, astrocytes become reactive by undergoing a range of morphological, functional, and phenotypic alterations, collectively called reactive astrogliosis [7, 8]. Previous research has detected two distinct types of reactive astrocytes (RAS) in accordance with their expression profile: neurotoxic A1-like astrocytes triggered by neuroinflammation, and neuroprotective A2-like astrocytes triggered by ischemia [9]. However, a recent study has pointed out that the defining subtypes such as A1 and A2 may not accurately reflect glial cell reactivity, and other new sub-states of astrocytes may be existed [10]. The above-mentioned studies have highly revealed the diversity and complexity of RAS. The A1-like astrocytes are triggered by activated microglia-released factors such as complement component 1q (C1q), interleukin-1 alpha (IL-1α), and tumor necrosis factor-alpha (TNF-α) and release a potent neurotoxin leading to impaired functioning of excitatory neurons in the CNS. Complement component 3 (C3) is considered to be the most characteristic marker of A1 subtype [9]. Besides, A1-like astrocytes are found abundantly in the brain of diverse human neurodegenerative diseases (NDDs), including Parkinson’s disease (PD), Huntington’s disease (HD), Amyotrophic lateral sclerosis (ALS), and Alzheimer’s disease (AD) [8, 11]. These astrocytes might be induced by the NF-κB pathway, showing strong association with neuroinflammation [12]. Therefore, the existence of A1-like astrocytes in human NDDs suggests that neuroinflammation might be contributing to or even driving neurodegeneration. Coincidentally, MDD patients have plentiful inflammatory traits, including elevated pro-inflammatory cytokines in the brain, cerebrospinal fluid, and peripheral blood, together with the neuroimaging visualization of reactive microglia [13]. Moreover, recent reports have demonstrated that the A1-like astrocytes are induced by lipopolysaccharide (LPS)-induced acute stress and chronic social defeat stress (CSDS) [14, 15]. Despite these findings, the significance of A1-like astrocytes in the pathogenesis and management of MDD still becomes uncertain.

As a two-pore-domain background K^+^ channel, TREK-1 regulates neuronal excitability apart from contributing to the background leak K^+^ currents [16]. TREK-1 is denoted in the entire CNS, especially the cortex, hippocampus, and hypothalamus [17]. It has a vital function in diverse CNS diseases, including ischemia [18], inflammation, pain [19], and depression [20]. TREK-1 deletion or suppression in the models of depression was investigated to be an efficacious antidepressant [21]. Recently, spadin, a sortilin-derived peptide, has been found to block TREK-1 with a high affinity and specificity, and suggested to inhibit approximately 60% of the TREK-1 current stimulated by arachidonic acid [22]. It also elicits a potent antidepressant action at merely 4 d post-injection [22]. So far, most studies associated with TREK-1 inhibitors have been carried out in neurons, and the mechanisms have been explained by elevated serotonin levels and improvement of neurogenesis and neuroplasticity [23]. However, astrocytes express TREK-1 in high amounts in many brain regions, and astrocytic TREK-1 is critical for passive conductance [24], and are involved in the homeostatic function of astrocytes, such as glutamate uptake and modulation of inflammation [25]. Nonetheless, the TREK-1 function associated with astrocyte reactivity has yet to be corroborated in MDD.

The current work intended to explore the effect of TREK-1 in the stimulation of A1-like reactive astrocytes in MDD, and to explicate the association of its mechanism with the NF-κB signaling pathway. We found that blocking TREK-1 by spadin mitigated the anxiety and depressive-like behaviors of rats under CUMS and compromised the A1 astrocytic reactivity in the hippocampus and astrocyte cell line of rats. We also demonstrated the implication of the NF-κB signaling pathway in the suppressive action of TREK-1 against the A1 astrocytic initiation. These findings illustrate that blockage of TREK-1 by spadin leads to the repression of A1-like reactive astrocytes in a rat model of MDD via the NF-κB signaling pathway. This study offers a novel idea for targeting astrocyte reactivity in MDD treatment.

## Materials and methods

### Animals

We procured male Sprague–Dawley (SD) rat with 150–180 g body mass from the Experimental Animal Center of Dalian Medical University (Dalian, China). Before experimentation, five rats were housed in every standard cage for 1 week at 23 ± 2 °C temperature under a 12-h light/12-h dark cycle and offered ad libitum feed and water. The present research protocol was based on the National Institutes of Health Guide for the Care and Use of Laboratory Animals and was approved under No: AEE19087 by the Animal Experiment Ethics Committee of the Dalian Medical University.

### Groups and drug administration

After one week of environmental adaptation and the baseline screening, 80 rats were classified into 4 groups (20 rats/group), namely CON (control group), CUMS (CUMS model group), CON + SPA (normal rats treated with spadin), and CUMS + SPA (CUMS-exposed rats treated with spadin) in a random manner. For 10 consecutive weeks, rats in CUMS and CUMS + SPA groups were subjected to various stressors. During 6–10 weeks, rats in CON + SPA and CUMS + SPA groups were intraperitoneally (i.p.) administered with normal saline or spadin (0.1 mg/kg, MedChemExpress, HY-P1422) once per day. The selection of spadin dosage was based on a prior study [26].

### Chronic unpredictable mild stress (CUMS) procedure

The CUMS procedure was implemented as reported previously with minor modifications [27]. The rats were isolated in individual cages as well as subjected to different stressors, as shown in Table 1. Daily, 1–2 varying stressors were imposed on these rats and were randomly administered over a week. However, the application of identical stressors on consecutive days was avoided.

**Table 1:** Schedule for CUMS procedure

Stressors	Durations
Water deprivation	24 h
Food deprivation	24 h
Overnight illumination	12 h
Cage titling (45°)	24 h
Physical restraint	1 h
Tail pinching	1 min
Wet bedding	24 h
Swimming in cold water (4 °C)	5 min
Empty bottle	12 h
Heat stress (45 °C)	5 min

### Sucrose preference test (SPT)

For the assessment of stress-induced anhedonia, a broadly practiced strategy named SPT was used. During the training phase, rats were acclimated to 2 bottles of sucrose solution (1%) ad libitum for an initial 24 h. One bottle of sucrose solution was replaced with an equivalent volume of potable water on the 2nd day. Next, the rats were deprived of food and water for a day in an independent cage. In the formal experiment, 2 identical bottles were provided to every rat, one involving sucrose solution (1%) and the other involving potable water. After 12 h, their positions were interchanged, and fluid consumption over 24 h was examined. The following computational equation for sucrose preference was utilized: Sucrose preference (%) = sucrose solution consumption/consumption of sucrose solution and water × 100%.

### Forced swimming test (FST)

Depressive-like behavior in rats was evaluated through FST. Briefly, each rat was placed in a 50 cm deep transparent Plexiglas cylinder with a diameter of 20 cm including water (23–25 °C) up to a height of 25 cm. After a training session, the formal test commenced where rats were individually forced to swim for 6 min. Following an initial 1 min phase of adaption, the immobility duration was monitored for 5 min. We videotaped every session for analytical purposes, and the water was replaced between the sessions. Any rat floating perpendicularly in the water or moving merely for living purposes by maintaining the head above the water surface was regarded as immobile.

### Open-field test (OFT)

In an open, brightly lit zone, the anxiety-like behavior and physical state of rats were examined through OFT using a 50 (L) × 50 (W) × 40 (H) cm bare square chamber. We placed each rat tenderly in the center of a black floor involving 16 peripheral and 9 central squares, a total of 25 equal squares with dimensions (10 cm × 10 cm). The time spent in the central region and complete activity path were recorded for 5 min with the aid of the Super Maze V2.0 video tracking system (Xinruan Technology, Shanghai). Between each session, alcohol (75%) was used to clean the chamber.

### Proteomic Analysis

The resultant peptides, after the collection, concentration, and trypsinization of hippocampal specimens, were labeled based on the instructions of the iTRAQ kit. Fractionation of tryptic peptides was done using a reverse-phase HPLC (high pH). In terms of LC–MS/MS analysis, the tryptic peptides were dissolved in 0.1% formic acid and then loaded directly onto a reversed-phase analytical column. They were eluted with solvent B (0.1% formic acid and 98% acetonitrile) on a UPLC system (EASY-nLC 1000). Thereafter, we subjected the peptides to tandem mass spectrometry (MS/MS) analysis in Q ExactiveTM Plus mass spectrometer (ThermoFisher Scientific) coupled online to the UPLC. Further, the Mascot engine (ver.1.5.2.8) was employed to assess the acquired data by searching the MS/MS spectra against the Uniprot_rat database. According to prior criteria, differential expressions of proteins were regarded as significant if the mean ratios were > 1.2 or < 0.83 and the p-values were < 0.05.

### Bioinformatics analysis

The recognized differentially expressed proteins (DEPs) were exposed to Gene ontology (GO) annotations including cellular component (CC), molecular function (MF), and biological process (BP), and the Kyoto Encyclopedia of Genes and Genomes (KEGG) database analysis. In addition, Fisher’s exact test (two-tailed) was utilized for the DEP abundance against entire recognized proteins. The corrected *p* < 0.05 was considered significant, and we transformed this filtered p matrix by -log10 (*p*).

### Western blotting

The rat hippocampal tissues and cultured astrocytes were collected, and western blotting was performed according to a prior procedure [28]. Briefly, the protein samples were isolated on 10–12% SDS-PAGE gel (30 µg per lane), and transferred onto polyvinylidene fluoride membranes (Millipore, USA). The membranes were exposed to incubation overnight at 4 °C with the primary antibodies shown in Table 2. An enhanced chemiluminescence technique was applied to assess the protein expression, while a ChemiDoc XRS system (Bio-Rad, USA) was utilized for imaging the blots. Additionally, the blot images were quantitatively assessed via Image J (National Institutes of Health, USA). GAPDH was applied to be the internal reference for data normalization.

**Table 2:** Primary antibodies used in experiments

Antibody	Host	Company	Cat.No	Application (Concentration)
TREK-1	Mouse	Santa Cruz	SC-398449	WB (1:1000)
C3	Rabbit	Proteintech	21337-1-AP	WB (1:1000)/IF (1:200)
IL-1α	Rabbit	Wanleibio	WL02541	WB (1:500)
TNF-α	Rabbit	Wanleibio	WL01581	WB (1:1000)
C1qA	Rabbit	Proteintech	11602-1-AP	WB (1:1000)
NF-κB p65	Rabbit	Bioss	bs-20355R	WB (1:1000)/IF (1:200)
P-NF-κB p65	Rabbit	Bioss	bs-3543R	WB (1:1000)/IF (1:200)
IκB-α	Rabbit	Wanleibio	WL01936	WB (1:500)
P-IκB-α	Rabbit	Wanleibio	WL02495	WB (1:500)
GAPDH	Mouse	Proteintech	60004-1-Ig	WB (1:5000)
IBA1	Rabbit	Proteintech	10904-1-AP	IF (1:200)
GFAP	Rabbit	Proteintech	16825-1-AP	IF (1:200)

### Quantitative Real-Time Polymerase Chain Reaction (qRT-PCR)

Trizol reagent (Takara, Japan) was adopted for the extraction of the total RNA from the cerebral tissues or cultured astrocytes of rats. Reverse transcription of the total RNA was done in accordance with the protocol of HiScript II Q RT SuperMix (Vazyme Biotech, China). The yielded cDNA was mixed with ChamQ Universal SYBR qPCR Master Mix (Vazyme Biotech, China) and gene-specific primers (Table 3) for a real-time PCR assessment in the CFX96 system (Bio-Rad, USA). We normalized the cycle time values according to the GAPDH internal control values. The − ΔΔCT (displayed in the heatmap) plus 2^−ΔΔCT^ (displayed in the histogram) method was employed for determining relative gene expressions.

**Table 3:** Gene-specifc primers used in the RT-qPCR analysis

Gene name	Forward primer (5'–3')	Reverse primer (5'–3')
GAPDH	GTGCCAGCCTCGTCTCATAG	AGAGAAGGCAGCCCTGGTAA
H2T23	ATGGAACCTTCCAGAAGTGGG	GAAGTAAGTTGGAGTCGGTGGA
Serping1	TGGCTCAGAGGCTAACTGGC	GAATCTGAGAAGGCTCTATCCCCA
H2-D1	ATGGAACCTTCCAGAAGTGGG	GAAGTAAGTTGGAGTCGGTGGA
Ggta1	TCTCAGGATCTGGGAGTTGGA	GAGTTCTATGGAGCTCCCGC
Ligp1	ATTTGGCTCGAAGCCTTTGC	ACGGCATTTGCCAGTCCTTA
Gbp2	TAAAGGTCCGAGGCCCAAAC	AACATATGTGGCTGGGCGAA
Fbln5	AGGGGGTTAAGCGAAACCAG	GTGAGTATCCTTTTAATCCTGGCA
Ugt1a	GGAAGCTGTTAGTGATCCCC	TGCTATGACCACCACTTCGT
Fkbp5	TGCAGTGTCGGCAGTTGTAT	GGGTCGCCCAAGTTAGAACA
Psmb8	TATCTGCGGAATGGGGAACG	AAAGTCCCGGTCCCTTCTTG
Srgn	GTTCAAGGTTATCCTGCTCGGA	AAACAGGATCGGTCATCGGG
Amigo2	GTTCGCCACAACAACATCAC	GTTTCTGCAAGTGGGAGAGC
Clcf1	GACTCGTGGGGGATGTTAGC	CCCCAGGTAGTTCAGGTAGG
Tgm1	AGACCCAATTTTCCTGGGGC	AGCGAGGACCTTCCATTGTG
Ptx3	CATCCCGTTCAGGCTTTGGA	CACAGGGAAAGAAGCGAGGT
S100a10	CATTTCACAGGTTTGCAGGGG	CCAGAGGGTCCTTTTGATTTTCC
Sphk1	AAAGCGAGACCCTGTTCCAG	CAGTCTGCTGGTTGCATAGC
Cd109	GTCGCTCACAGGTACCTCAA	CTGTGAAGTTGAGCGTTGGC
Ptgs2	CTCAGCCATGCAGCAAATCC	GGGTGGGCTTCAGCAGTAAT
Emp1	ACCATTGCCAACGTCTGGAT	TGGAACACGAAGACCACGAG
Slc10a6	TCCATAGAGACCGGAGCACA	ATGCCTGATATGCTGCGACA
Tm4sf1	CTGAGGGACAGTACCTTCTGGATTC	GGCTAGGCCTCAACACAGTTA
B3gnt5	TGCTCCTGGATGAAAGGTCC	ACATGCTTGATCCGTGTGGT
Cd14	TCAGAATCTACCGACCATGAAGC	GGACACTTTCCTCGTCCTGG
C3	TTGTCCCCTTGAAGATCGGC	TCATTCCTTCTGGCACGACC

### Immunofluorescence staining

Immunofluorescence staining was performed according to our prior procedure [28]. Briefly, the cerebral tissues of rats were embedded in OCT and sliced by a cryosectioning machine (Leica, Germany) into coronal sections of 20 µm thickness. Cultured astrocytes were immobilized with paraformaldehyde (4%). Entire samples were blocked with goat serum albumin (10%) and overnight incubation of the samples at 4 °C was carried out using the primary antibodies shown in Table 2. Subsequently, the samples were incubated with FITC Goat Anti-Mouse IgG or Cy3 Goat Anti-Rabbit IgG (1:200, ABclonal, China) secondary antibodies, followed by the DAPI staining (Solarbio, China). A fluorescence microscope (Leica, Germany) was utilized for the imaging, and Image J was employed for the image analysis.

### Astrocyte culture and treatment

The immortalized rat astrocyte cell line CTX-TNA2 (OtwoBiotech, Shenzhen, China) were cultivated in Dulbecco’s modified Eagle’s medium/F12 medium (DMEM/F12) (KeyGEN, Jiangsu, China) with 10% concentration of fetal bovine serum (FBS) (Gibco, USA), and kept at 37 °C in 5% CO_2_. CTX-TNA2 cells were plated in 25 cm^2^ flasks and fed every 1–2 days. According to a prior procedure [9], CTX-TNA2 astrocytes were stimulated with ACM containing 400 ng/mL C1q (Novus protein, NBP2-62,410), 3 ng/mL IL-1α (Peprotech, 400–01), and 30 ng/mL TNF-α (CST, 8902) for 24 h to induce A1-like astrocytes. To determine the dosage of spadin, CTX-TNA2 astrocytes were subject to treatment with various dosages of spadin (0.01, 0.05, 0.1, 1, and 10 µmol/L) for 24 h with or without ACM induction. In addition, TREK-1 agonist BL-1249 (10 µM, MedChemExpress, HY-108596) was administrated for 24 h following ACM induction as described previously [29]. NF-κB inhibitor pyrrolidine dithiocarbamic acid (PDTC) (50 µM, MedChemExpress, HY-18738) was added at 1 h before ACM induction as per the manufacturer’s protocol and a prior procedure [30].

### Cell counting kit-8 (CCK8) assay

Based on the instructions of a CCK8 kit (Beyotime, Shanghai, China), CCK8 assay was carried out. Briefly, a density of 10^5^ cells/mL was seeded in 96-well microplates followed by the supplementation of spadin and ACM in varying concentrations. The microplates were added with CCK8 reagent (10 µL/well) and then read with a spectrophotometer to identify the absorbance at 450 nm.

### Statistical analysis

The data were investigated statistically with the use of Prism ver.9.0 (GraphPad Software, La Jolla, CA) and are shown to be means ± standard errors of the means (SEMs) from a minimum of triplicate tests. Student’s t-test was adopted for making the two-sample comparisons, while one-way analysis of variance (ANOVA) and subsequent Tukey’s post hoc test were adopted with the aim of carrying out multiple-sample comparisons. *p* < 0.05 was thought to be of statistical significance.

## Results

### Blocking TREK-1 significantly ameliorates CUMS-mediated depressive-like behaviors in rats

**Fig. 1:**
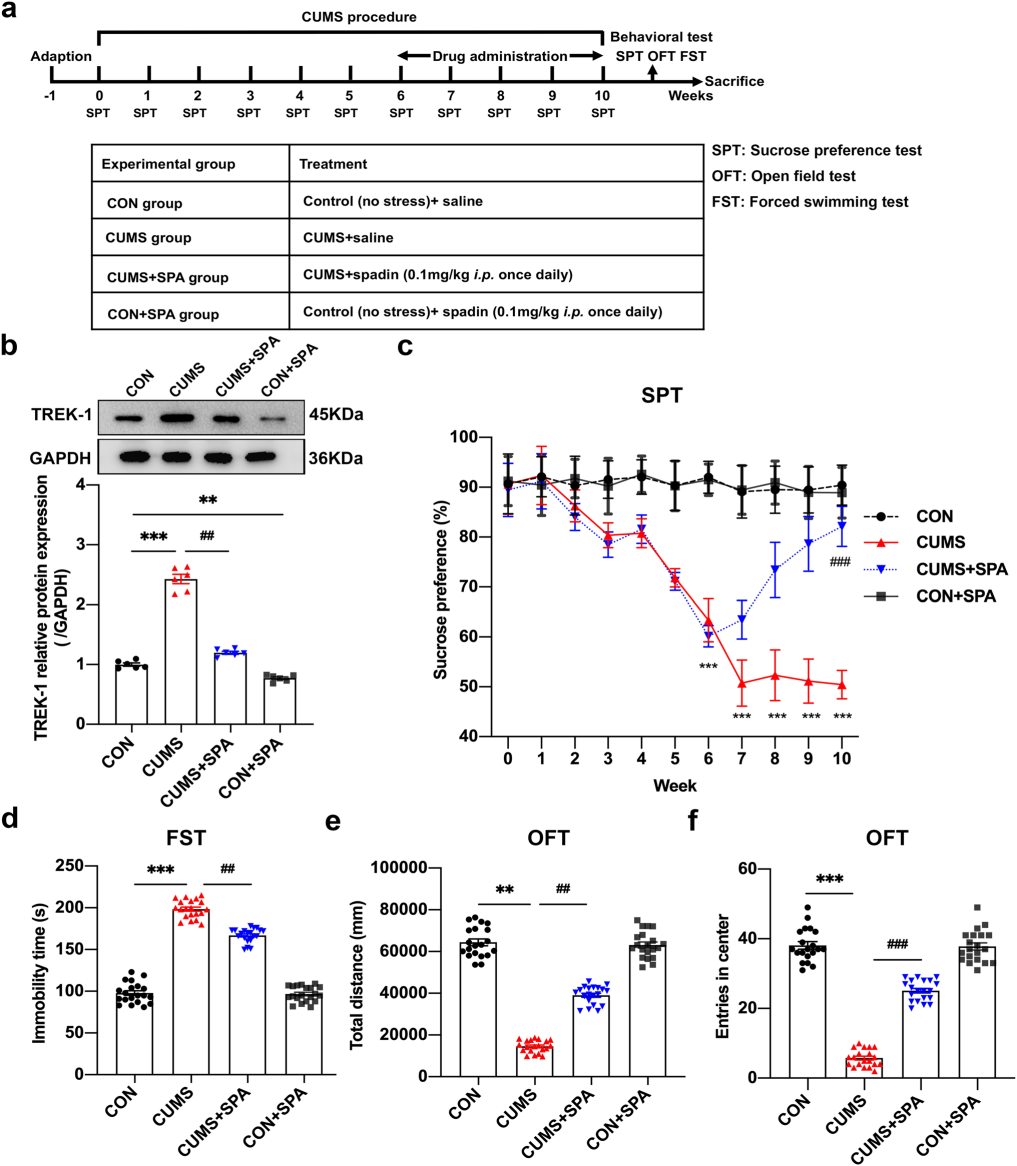
TERK-1 specific blocker spadin alleviated depressive-like behaviors in CUMS rats. **a** Timeline for the experimental design. **b** Representative western blot of TREK-1 expression in the hippocampus. **c** Sucrose preference percentage within 10 weeks. **d** Immobility time from FST. **e** Overall movement distance from OFT. **f** Entry number in the central region of the OFT. Dates can be indicated as mean ± SEM. **b** n = 6/group, **c–f** n = 20/group, **p < 0.01 and ***p < 0.001 vs. CON group; ^##^*p* < 0.01 and ^###^*p* < 0.001 vs. CUMS group

With the purpose of confirming the function of TREK-1 in the pathogenesis of MDD in vivo, spadin was used to inhibit TREK-1 expression. The steps followed in the experiment are depicted in Fig. 1a. The western blotting results indicated that hippocampal expression of TREK-1 was elevated in the CUMS group in comparison with the CON group (Fig. 1b, *p* < 0.001), while spadin administration reduced the CUMS-induced high expression of TREK-1 (*p* < 0.01). After 6-week CUMS stimulation, the sucrose preference rate was significantly reduced (Fig. 1c, *p* < 0.001), which was enhanced by spadin treatment for 4 weeks (*p* < 0.001). Immobility time in rats was found to be notably elevated in FST after CUMS stimulation (Fig. 1d, *p* < 0.001). However, spadin treatment could reverse this impact (*p* < 0.01). According to OFT experiments, total moving distance (Fig. 1e, *p* < 0.01) along with the number of entries in center (Fig. 1f, *p* < 0.001) were reduced in CUMS rats, which were notably increased by spadin (*p* < 0.01,* p* < 0.001, respectively). In line with the obtained results, TREK-1 blocking significantly ameliorated depressive-like behaviors in CUMS rats.

### Functions of TREK-1 blocking in the hippocampal proteomics of CUMS rats

**Fig. 2:**
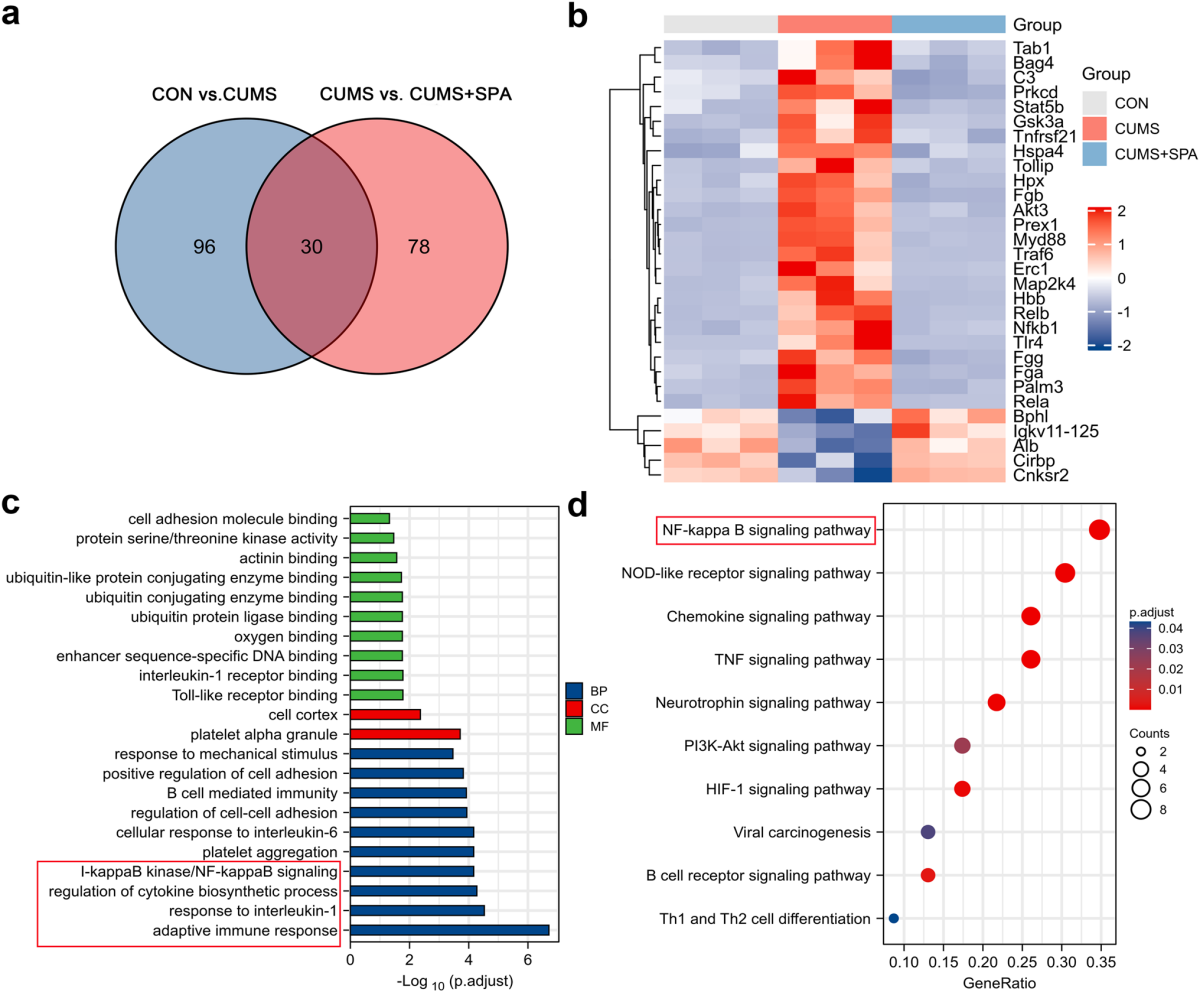
Role of TREK-1 blocking in the hippocampal proteomics within CUMS rats. **a** The number of overlapped DEPs in CON vs. CUMS and CUMS + SPA vs. CUMS were summed up using a Venn diagram. **b** Heatmap of the overlapped DEPs. **c** GO enrichment of the overlapped DEPs. **d** KEGG pathway analysis of the overlapped DEPs

To analyze the molecular mechanism associated with the anti-depressive effect of TREK-1, quantitative proteomics was performed on the hippocampus samples of rats. We detected a total of 5437 proteins and were able to quantify 4458 proteins. By using the threshold values of up-regulated proteins with *p* < 0.05 and quantification ratio > 1.2, and of down-regulated proteins with *p* < 0.05 and quantification ratio < 0.83, a total of 204 DEPs were obtained. As shown in Fig. 2a, 126 DEPs in CUMS vs. CON group and 108 DEPs in CUMS vs. CUMS + SPA group were identified. In addition, 30 overlapping DEPs were identified in both CUMS vs. CON group and CUMS + SPA vs. CUMS group. The heatmap analysis of the overlapping DEPs showed that 25 proteins presented up-regulation and 5 proteins presented down-regulation in CUMS group in relative to the CON group, whereas spadin treatment was able to reverse these effects in all of the overlapping DEPs (Fig. 2b). Moreover, GO/KEGG pathway analysis for examining overlapping DEPs identified the BP enriched in adaptive immune response, response to interleukin-1 and I-κB kinase/NF-κB pathway, etc. The DEPs were found to be associated with the MF of Toll-like receptor binding and interleukin-1 receptor binding, etc. (Fig. 2c). Based on the KEGG database, the DEPs were mostly enriched in NF-κB, TNF, NOD-like receptors and chemokine signaling pathways, etc. (Fig. 2d). Based on the obtained findings, the potential molecular mechanism in association with the antidepressant action of TREK-1 may be significantly related to neuroinflammatory response and NF-κB signaling pathway.

### Blocking TREK-1 hinders microglia activation and lowers the hippocampal expression of C1q, TNF-α, and IL-1α in CUMS rats

**Fig. 3:**
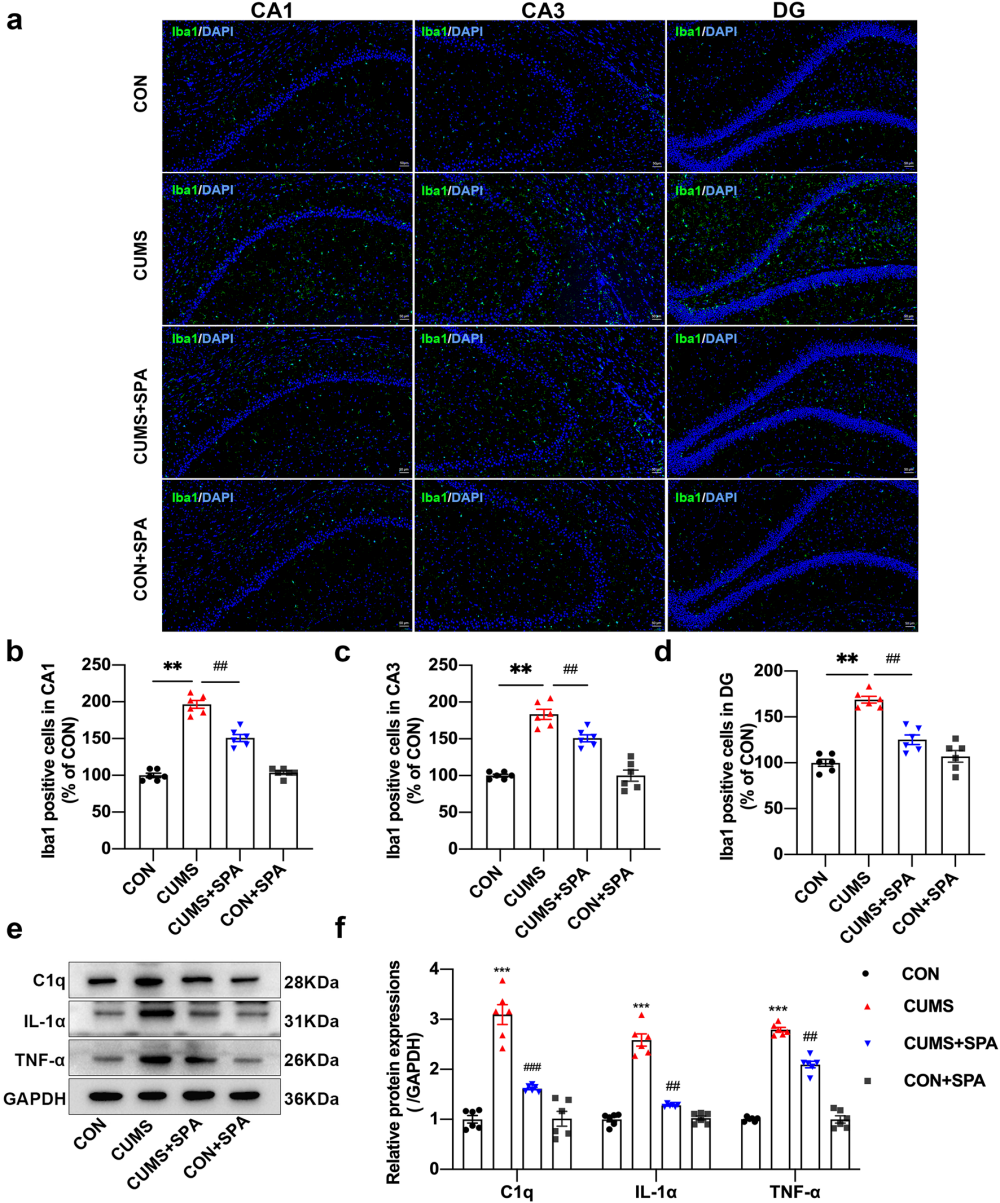
Role of TREK-1 blocking in microglia activation and C1q, IL-1α, and TNF-α levels in the hippocampus of CUMS rats. **a** Immunofluorescent images showing Iba1-positive microglia (green) in CA1, CA3, and DG hippocampal regions. Scale bar = 50 µm. **b–d** The number of Iba1-positive cells in the above three regions. **e**, **f** Western blot of hippocampal TNF-α, C1q, and IL-1α protein expressions. Dates are shown to be mean ± SEM. n = 6 per group, **p < 0.01 and ***p < 0.001 vs. CON group; ^##^*p* < 0.01 and ^###^*p* < 0.001 vs. CUMS group

Microglia have been documented to be the primary source of inflammatory cytokine generation during the early neuroinflammatory response phase, whereas astrocytes exert a vital function during the late chronic inflammatory response phase [31]. Thus, we first attempted to explore the function of TREK-1 on the activation of microglia and inflammatory cytokines in CUMS rats. We performed immunolabeling of microglia as depicted in Fig. 3a and found that Iba1 expression was notably elevated in CA1, CA3, and DG hippocampal regions of CUMS rats (Fig. 3b–d, all *p* < 0.01). However, a significant reduction in Iba1 expression was observed in these regions of rats after treatment with spadin (all *p* < 0.01). Further, C1q, IL-1α, and TNF-α protein expressions were discovered to be markedly elevated in the CUMS group compared to the CON group (Fig. 3e, f, all *p* < 0.001). However, spadin treatment notably lowered the levels of C1q (*p* < 0.001), IL-1α (*p* < 0.01), and TNF-α (*p* < 0.01). The findings indicated that TREK-1 blocking could inhibit the activation of microglia and the expression of C1q, IL-1α, and TNF-α in the hippocampus of CUMS rats, which might suppress the induction of A1-like reactive astrocytes.

### Blocking TREK-1 suppresses A1-like astrocyte reactivity in the hippocampus of CUMS rats

**Fig. 4:**
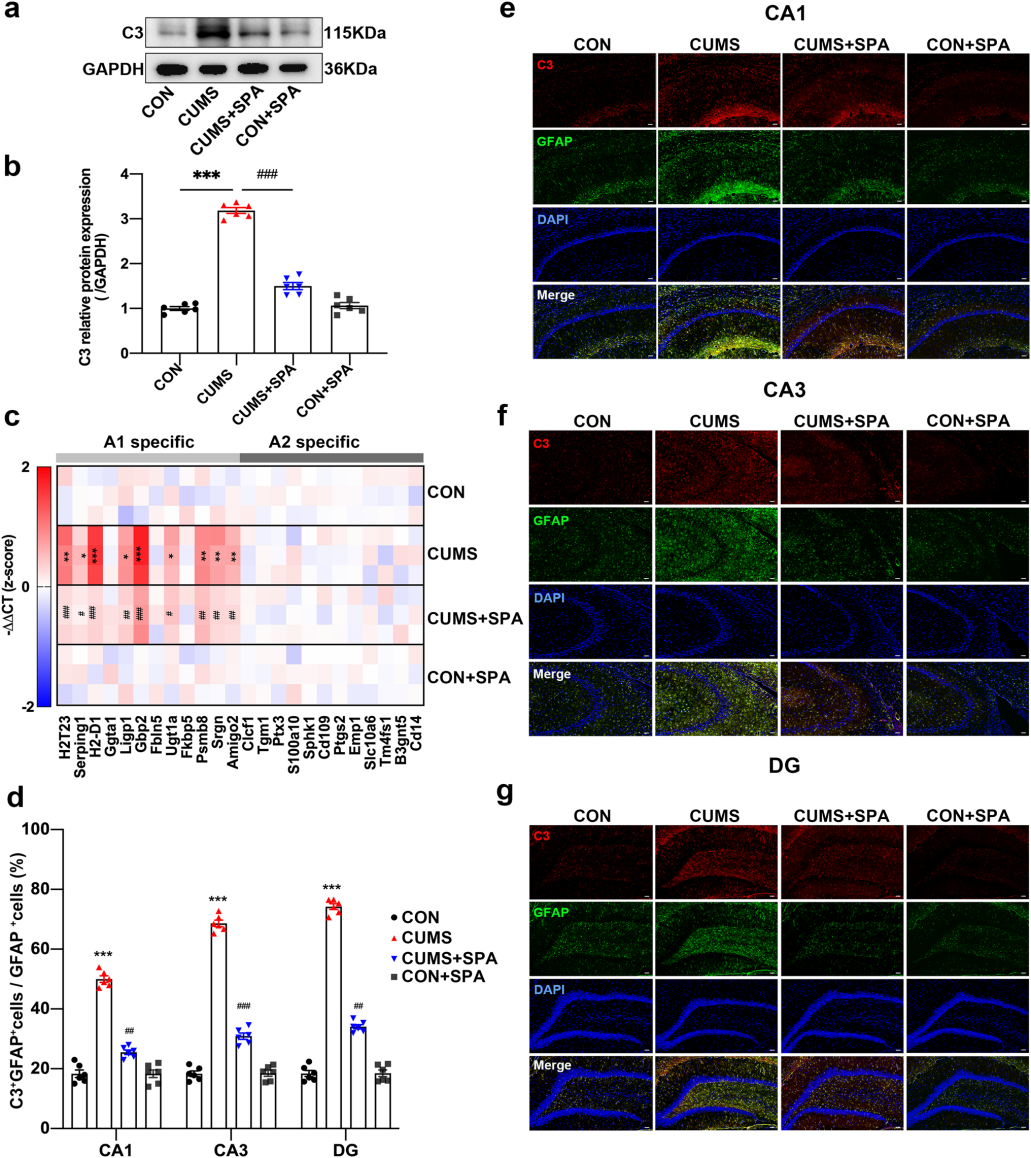
Impact of TREK-1 blocking on A1-like astrocyte activation in the hippocampus of CUMS rat. **a**, **b** Western blot of C3 protein in rat hippocampus. **c** Heatmap showing A1- and A2-specific transcripts in the hippocampus according to RT-qPCR. **d** Quantification of C3^+^/GFAP^+^ cells in CA1, CA3, and DG hippocampal regions. **e–g** Immunofluorescent images showing C3 (red) and GFAP (green) positive astrocytes in the above regions. Scale bar = 50 µm. Dates are shown to be mean ± SEM. n = 6/group, *p < 0.05, **p < 0.01 and ***p < 0.001 vs. CON group; ^#^*p* < 0.05, ^##^*p* < 0.01and ^###^*p* < 0.001 vs. CUMS group

To investigate the impact of TREK-1 on reactive astrocytes in the hippocampus of CUMS rats, the specific markers of A1 and A2 were detected. The findings showed that the C3 protein level was notably raised in the hippocampus of CUMS rats (Fig. 4a, b, *p* < 0.001), whereas it was lowered significantly by spadin treatment (*p* < 0.001). The mRNA levels of A1-specific markers were discovered to be elevated in hippocampus of CUMS rats (Fig. 4c, *p* < 0.001 for H2-D1, Gbp2; *p* < 0.01 for H2T23, Psmb8, Srgn, and Amigo2; *p* < 0.05 for Serping1, Ligp1, and Ugt1a). However, the elevated effects were reversed by spadin treatment (*p* < 0.001 for H2T23, H2-D1, Gbp2; *p* < 0.01 for Ligp1, Psmb8, Srgn, and Amigo2; *p* < 0.05 for Serping1, and Ugt1a). On the contrary, CUMS treatment, as well as spadin exposure, did not influence the transcript levels of A2-specific markers in rat hippocampus. These data demonstrated a strong relationship between TREK-1 and the transformation of A1-like astrocytes but not A2-like astrocytes. In addition, double immunofluorescence labeling of GFAP and C3 in CA1, CA3, and DG hippocampal regions was performed and shown in Fig. 4e–g. These data revealed an increasing number of C3 and GFAP double-positive cells in the hippocampal regions of CUMS rats in relative to the control (Fig. 4d, all *p* < 0.001). Remarkably, this increase in C3^+^GFAP^+^ cell number was decreased by spadin treatment in CA1 (*p* < 0.01), CA3 (*p* < 0.001) as well as DG (*p* < 0.01) hippocampal regions. Moreover, the obtained findings suggested that blocking TREK-1 decreased A1-like astrocyte reactivity in CUMS rats, which might contribute to its novel antidepressant mechanism.

### Blocking TREK-1 hinders the CUMS-induced NF-κB activation in the hippocampus of rats

**Fig. 5:**
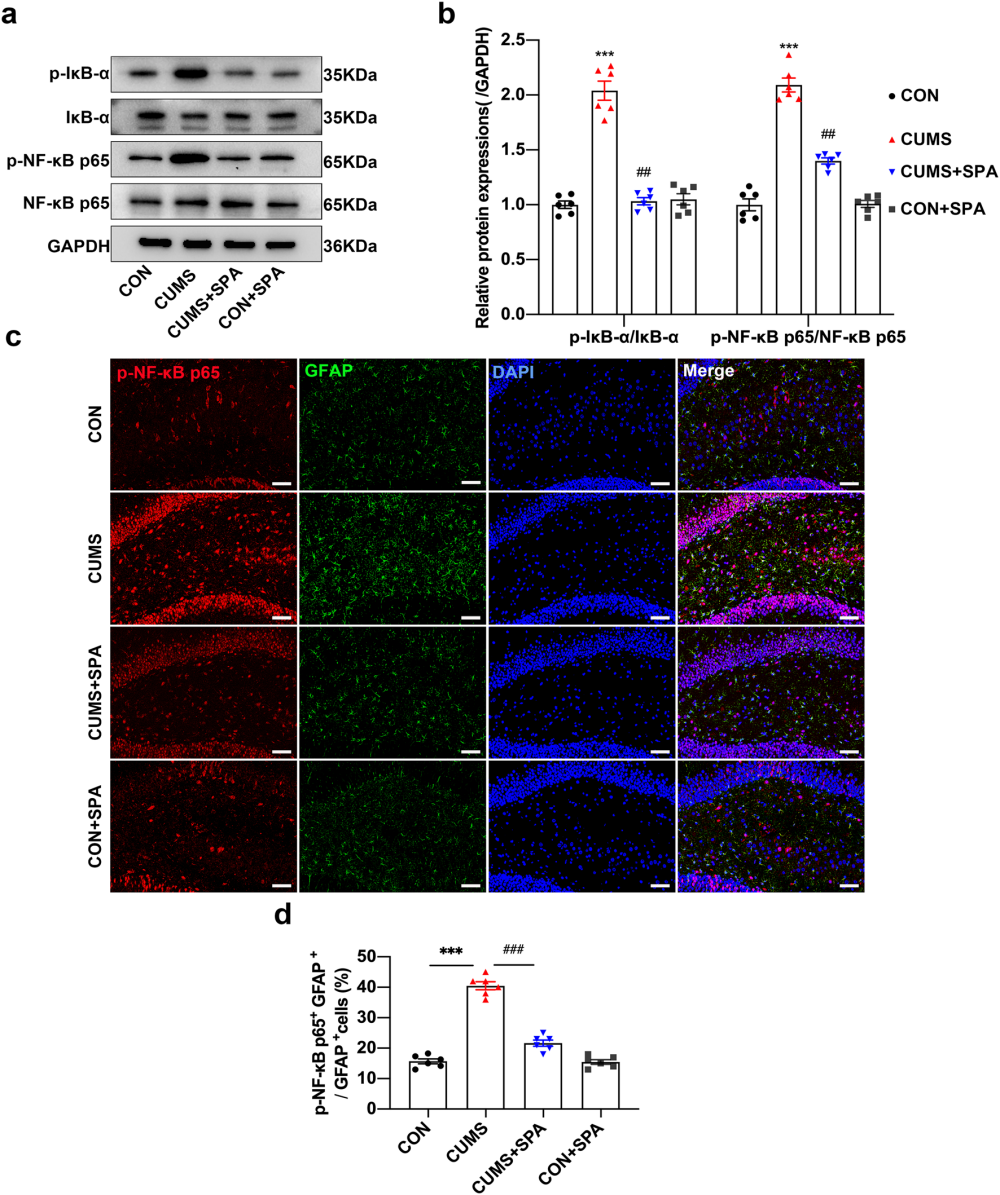
Role of TREK-1 blocking in the CUMS-induced NF-κB pathway in rat hippocampus. **a**, **b** Western blot of p-IκB-α, total IκB-α, p-NF-κB p65, and total NF-κB P65 protein levels in the hippocampus of rats. The immunoreactive bands for phosphorylated protein were subjected to normalization with the unphosphorylated one. **c** Immunofluorescence images for p-NF-κB p65 (red) and GFAP (green) colocalization in the hippocampal DG region. Scale bar = 20 µm. **d** p-NF-κB p65^+^/GFAP ^+^ cells quantified in the hippocampal DG region. Dates are shown to be mean ± SEM. n = 6/group, **p < 0.01 and ***p < 0.001 vs. CON group; ^##^*p* < 0.01 and ^###^*p* < 0.001 vs. CUMS group

Based on proteomics, we further verified the impact of TREK-1 on the NF-κB pathway in vivo. The phosphorylation levels of IκB-α and NF-κB p65 in the hippocampus were increased considerably in CUMS-treated rats compared to control rats. (Fig. 5a, b, all *p* < 0.001). Moreover, spadin treatment notably lowered these phosphorylation levels (all *p* < 0.01). Next, we adopted double immunofluorescence staining to evaluate p-NF-κB p65 levels within hippocampal astrocytes (Fig. 5c). The p-NF-κB p65 levels in the astrocytes of CUMS rats were significantly elevated (Fig. 5d, *p* < 0.001), while spadin treatment led to a decrease p-NF-κB p65 expression compared with the CUMS group (*p* < 0.001). The findings showed that blocking TREK-1 could inhibit CUMS-induced NF-κB pathway activation in the astrocytes of rat hippocampus, suggesting the involvement of the NF-κB pathway in TREK-1-associated inhibition of A1-like astrocytes.

### Blocking TREK-1 attenuates the production of A1-like astrocytes in vitro

**Fig. 6:**
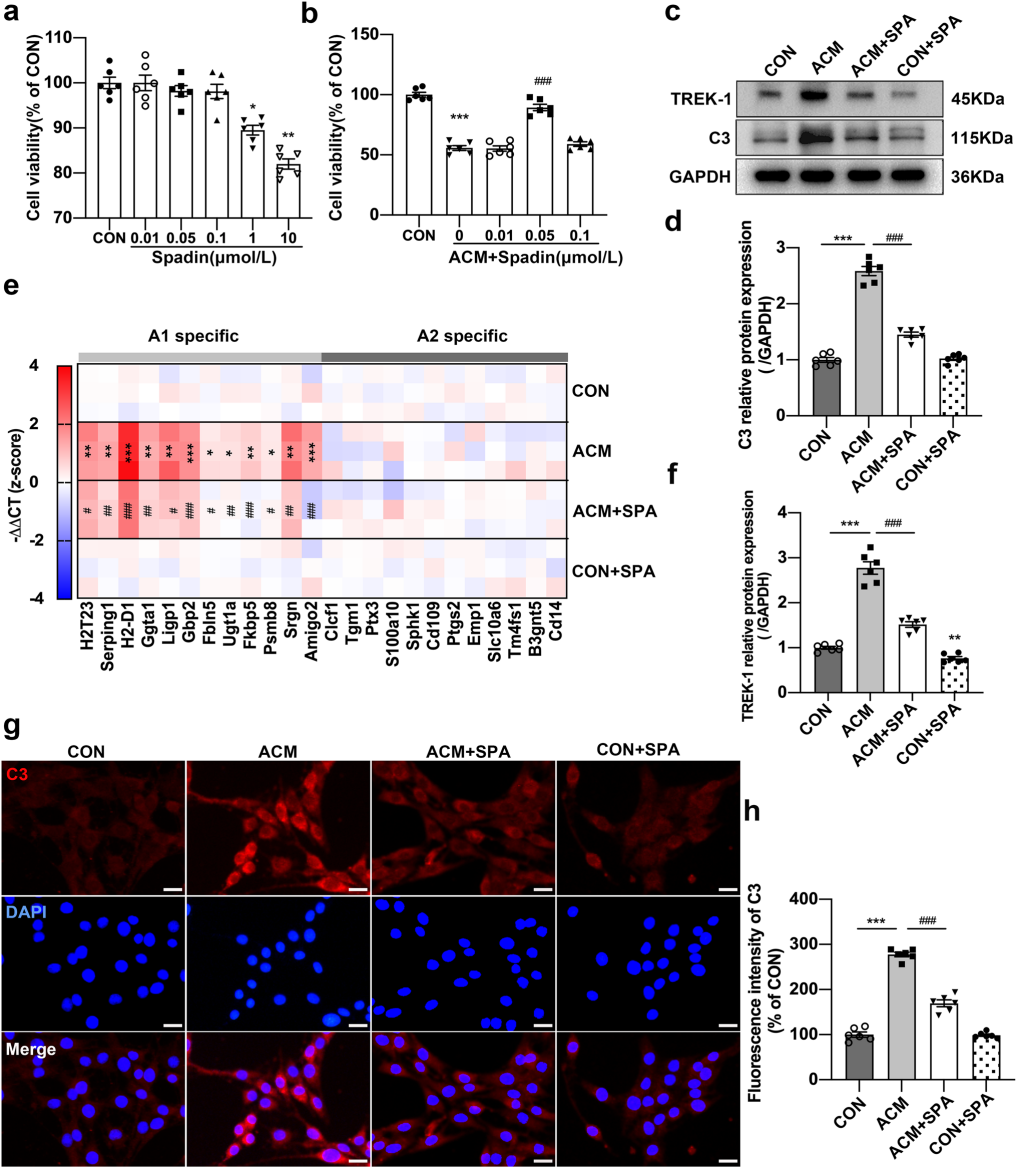
Role of TREK-1 blocking in A1-like astrocyte activation in vitro. **a** Astrocyte viability upon various dosages administration of spadin for 24 h. **b** Astrocyte viability upon various dosages administration of spadin with/without ACM for 24 h. **c**, **d**, **f** Western blot of TREK-1 and C3 protein expressions after ACM and spadin treatments. **e** Heatmap showing A1- and A2-specific transcripts within astrocytes following ACM stimulation and spadin treatment. **g** Immunofluorescence images presenting C3 (red) and DAPI (blue) after ACM and spadin treatments. Scale bar = 50 µm. **h** Quantitative analysis of C3 immunofluorescence intensity. Dates are denoted to be mean ± SEM. n = 6/group, *p < 0.05, **p < 0.01 and ***p < 0.001 vs. CON group; ^#^*p* < 0.05, ^##^*p* < 0.01 and ^###^p < 0.001 vs. ACM group

We chose the rat astrocyte cell line CTX-TNA2 to explore the role of TREK-1 in A1-like astrocyte transition in vitro. Firstly, the impact of spadin on the astrocyte viability was identified by measuring the optimal spadin content by adopting the CCK8 assay. CTX-TNA2 astrocytes were exposed to variable concentrations (0.01, 0.05, 0.1, 1, and 10 µmol/L) of spadin for 24 h, and the results showed that spadin concentrations of 1 and 10 µmol/L generated an obvious reduction in cell viability (Fig. 6a, *p* < 0.05, *p* < 0.01, respectively), while the concentrations less than 1 µmol/L did not show any influence on the cell viability. The CTX-TNA2 cells were stimulated with ACM for 24 h to induce A1-like astrocytes. We further treated A1-like astrocytes with 0.01, 0.05, and 0.1 µmol/L of spadin for 24 h, and the CCK-8 assay showed that 0.05 µmol/L spadin significantly reversed the ACM-induced reduction in cell viability of astrocytes (Fig. 6b, *p* < 0.001). Based on these results, 0.05 µmol/L spadin was used in further experiments. The results of western blotting shown in Fig. 6c verified that the protein levels of TREK-1 and C3 were notably elevated in ACM-induced astrocytes (Fig. 6d, f, all *p* < 0.001), which were reversed by spadin treatment (all *p* < 0.001). In addition, spadin treatment decreased mRNA expression of A1-specific markers triggered by ACM (Fig. 6e, ACM vs. CON *p* < 0.001 for H2-D1, Gbp2, and Amigo2, *p* < 0.05 for Fbln5, Ugt1a, and Psmb8, *p* < 0.01 for other markers; ACM + SPA vs. ACM *p* < 0.001 for H2-D1, Gbp2, Fkbp5, and Amigo2; *p* < 0.01 in Serping1, Ggta1, Ugt1a, and Srgn, *p* < 0.05 for additional markers), while ACM and spadin administration generated no impacts on A2-specific transcript levels (Fig. 6e). Furthermore, we measured changes in C3 immunofluorescence of astrocytes treated with ACM and spadin (Fig. 6g). Our data showed a significant increase in the C3 immunofluorescence intensity after ACM stimulation (Fig. 6h, *p* < 0.001), while spadin treatment resulted in the inhibition of this increase (*p* < 0.001). The findings suggested that TREK-1 expression was significantly increased in A1-like astrocytes, while blocking TREK-1 hindered A1-like astrocyte activation in vitro.

### Blocking TREK-1 suppresses A1-like astrocyte activation via* the NF-κB pathway *in vitro

**Fig. 7:**
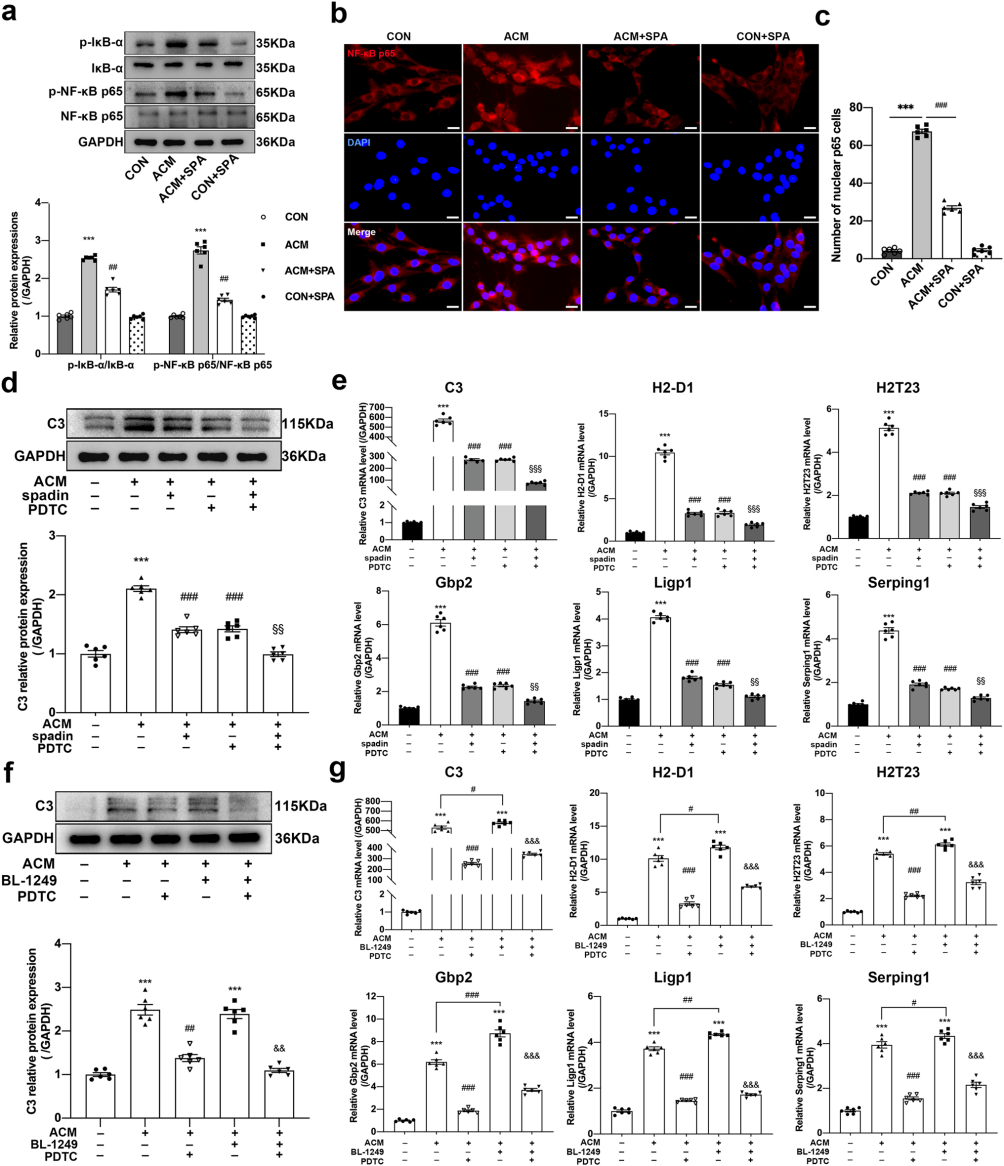
NF-κB signaling pathway participation in the impacts of TREK-1 on A1-like astrocyte activation in vitro. **a** Western blot of p-IκB-α, total IκB-α, p-NF-κB p65, and total NF-κB p65 protein levels in ACM-stimulated astrocytes. Immunoreactive bands for phosphorylated protein were exposed to normalization with the unphosphorylated one. **b** Immunofluorescent images presenting DAPI (blue) and NF-κB p65 (red) in ACM-stimulated astrocytes. Scale bar = 50 µm. **c** Quantification of NF-ĸB p65 immunofluorescence intensity in the nucleus. **d** Western blot analysis of C3 expression in ACM-stimulated astrocytes treated with spadin (0.05 µM, 24 h), PDTC (50 µM, 1 h) or their combination. **e** RT-qPCR showing relative mRNA levels of A1-specific transcripts in the astrocytes of different groups as indicated. **f** Western blot analysis of C3 expression in ACM-stimulated astrocytes treated with BL-1249 (10 µM, 24 h), PDTC or their combination. **g** RT-qPCR showing the relative mRNA levels of A1-specific transcripts in the astrocytes of different groups as indicated. Data are expressed as mean ± SEM. n = 6/group, ***p < 0.001 vs. CON group; ^#^*p* < 0.05, ^##^*p* < 0.01 and ^###^*p* < 0.001 vs. ACM group; ^§§^*p* < 0.01 and ^§§§^*p* < 0.001 vs. ACM + spadin group; ^&&^*p* < 0.01 and ^&&&^*p* < 0.001 vs. ACM + BL-1249 group

Western blotting was conducted to analyze the impacts of spadin treatment on IκB-α and NF-κB p65 phosphorylation within ACM-induced astrocytes. We found that ACM stimulation caused enhancement in p-IκB-α and p-NF-κB p65 levels (Fig. 7a, all *p* < 0.001). On the contrary, treatment with spadin (0.05 µM, 24 h) significantly decreased their phosphorylation levels (all *p* < 0.01). Further, the immunofluorescence staining demonstrated that ACM stimulation resulted in NF-κB p65 translocation (Fig. 7b, c, *p* < 0.001), while spadin administration hindered this translocation (*p* < 0.001). Next, the NF-κB inhibitor, PDTC, was adopted to reveal the role of NF-κB pathway in the effect of TREK-1 on A1-like astrocytes. The CCK-8 and western blot results showed that a pretreatment with 50 µM PDTC for 1 h markedly decreased p-P65 protein levels (Fig. S1). Moreover, PDTC significantly diminished the high expressions of A1-specific markers induced by ACM (Fig. 7d, e, ACM vs. CON all *p* < 0.001; ACM + PDTC vs. ACM all *p* < 0.001), and exhibited a stronger synergistic effect on the down-regulation of C3 (Fig. 7d, *p* < 0.01) and A1-specific transcripts (Fig. 7e, *p* < 0.001 for C3, H2-D1, and H2T23; *p* < 0.01 for Ligp1, Gbp2, and Serping1) when it was combined with spadin. In addition, treatment with BL -1249 (a TREK -1 agonist, 10 µM, 24 h) further promoted ACM-induced mRNA levels of A1-specific transcripts (Fig. 7g, ACM + BL-1249 vs. ACM *p* < 0.05 for C3, H2-D1 and Serping1; *p* < 0.01 for H2T23 and Ligp1; *p* < 0.001 for Gbp2). However, pretreatment with PDTC partially abolished the effects of BL-1249 on the activation of A1-like astrocytes (Fig. 7f, *p* < 0.01; Fig. 7g, all *p* < 0.001). In summary, the NF-κB signaling pathway can be deemed to be necessary for the effects of TREK-1 on A1-like astrocyte activation.

## Discussion

The present study demonstrated that blocking TREK-1 alleviated depression and anxiety behaviors in rats and hindered microglia activation and A1-like astrocyte within the rat hippocampus using the CUMS rat model of MDD. To clarify the underlying mechanisms of TREK-1, this study carried out in vivo and in vitro molecular biology assays, finding that the NF-κB signaling pathway played vital function in the effect of TREK-1 on A1-like astrocyte activation in MDD. Together, these findings provide the first evidence that blocking TREK-1 by spadin inhibited the A1-like astrocyte activation by modulating the NF-κB signaling pathway.

TREK-1 is regarded as a viable therapeutic target in treating depression [32]. Chronic suppression of TREK-1 can protect from chronic stress-mediated depression as well as hippocampal synaptogenesis impairment in mice [21]. In recent years, spadin has been discovered as a novel antidepressant that exerts rapid and effective action by selectively blocking the TREK-1 channel [33]. Spadin refers to a modified version of NeuroTenSinReceptor-3 (NTSR3) /sortilin propeptide, which is released into the bloodstream after being cleaved by the protein convertase furin [22]. Neurotensin (NT) receptor has been reported to interact with the TREK-1 channel physically and control its expression on the cell surface, showing a high affinity for spadin [34]. A previous study showed that in the same models of depression, animals exposed to spadin treatment acted identically to the TREK-1 knockout mice or fluoxetine-treated mice [22]. Spadin has the advantages of high specificity, rapid onset of action, long lasting and stable action, readily crossing the blood brain barrier, and no adverse effects on TREK-1-controlled functions [35].Therefore, we used spadin to block TREK-1 in rats and found that 4-week spadin treatment prominently downregulated the expression of TREK-1 and distinctively lowered depressive-like behaviors that were measured by SPT, FST, and OFT in CUMS exposed rats, conforming to the above-mentioned reports. Moreover, the antidepressant mechanisms of TREK-1 have been focused on neurons and illustrated by an increased amount of serotonin in the brain based on enhanced neuronal activity [36]. Nevertheless, the antidepressant effects of TREK-1 on glial cells are still unclear. Hence, to deeply investigate the underlying antidepressant mechanisms of TREK-1, this study performed the proteomic analysis on the hippocampus samples of CUMS rats. The most obvious finding was that the DEPs could be notably enhanced in the processes associated with neuroinflammation and stress response. Microglia have been reported to be the major immune effector cells and play a fundamental role in depression-related inflammation [37]. As discovered in a previous study, microglia deficiency before etomidate treatment hinders etomidate-mediated activation of A1 reactive astrocytes [38]. Based on this finding, we subsequently investigated the impacts of TREK-1 on microglia activation. Interestingly, we found that blockage of TREK-1 significantly inhibited microglia activation as well as the inflammatory factor production of IL-1α, TNF-α, and C1q in the hippocampus of CUMS rats. It indicated that blocking TREK-1 might further reverse the induction of A1-like reactive astrocytes.

The formation of reactive A1 astrocytes is a basic pathological response to numerous neurodegenerative diseases. A1-like astrocytes generate a strong neurotoxic impact and amplifies inflammatory microglial responses [39]. Moreover, A1-like astrocytes are less capable of promoting the formation of new synapses, causing a reduction in the excitatory function of CNS neurons [40]. In recent studies, A1-like astrocytes are associated with depressive behaviors triggered via multiple animal models of depression [14, 15, 41]. However, the exact mechanism and intervention of A1-like astrocyte in MDD are still lacking. The presented in vivo data revealed that blockage of TREK-1 by spadin decreased the levels of specific A1 markers in different hippocampal regions of CUMS rats. Furthermore, the in vitro findings demonstrated that spadin treatment could block A1-like astrocyte activation following ACM stimulation. Overall, the decreased reactivity of A1-like astrocyte might contribute to the antidepressant impact of spadin in vivo* and *in vitro. The regulators of the A1-like astrocytes may become the therapeutic targets to treat MDD.

NF-κB refers to a vital transcription factor in regulating neuroinflammatory responses [42]. It exerts a vital function in MDD, and NF-kB hyperactivity generates pro-inflammatory factor, which represents a key driver in the clinical manifestation of depressive symptoms [28]. Additionally, previous studies have found that the astrocytic NF-κB activation can stimulate the generation of the characteristic A1 marker C3 through directly binding to its promoter in AD [12]. By contrast, the NF-κB inhibitor treatment inhibits the generation of C3 in A1-like astrocytes triggered by unconjugated bilirubin (UCB) [43]. These findings indicate that astrocytic NF-κB activation may drive astrocytes to acquire the A1 phenotype and exert a vital function in astrocyte-mediated neuroinflammation. NF-κB is commonly maintained in the cytoplasm through its interaction with IκB. after stimulation, IκBα decomposition and phosphorylation are essential for p65 phosphorylation and nuclear translocation of NF-κB [44]. Our in vivo data found that blockage of TREK-1 inhibited the increased protein expressions and fluorescence intensity of p-NF-κB p65 in the hippocampal of CUMS-exposed rats. These findings confirmed the proteomic analysis. To deeply explore whether NF-κB pathway was related to the impacts of TREK-1 on activation of A1-like astrocytes, we further carried out in vitro experiments. The results confirmed that blockage of TREK-1 led to decreased phosphorylation of NF-κB p65 and inhibited p65 nuclear translocation in astrocytes after ACM stimulation. Interestingly, we also confirmed that PDTC, an inhibitor of NF-κB signaling pathway, significantly hindered the activation of A1-like astrocytes, and more importantly, the inhibitory effects are more significant if spadin and PDTC were applied at the same time. Furthermore, we found that the administration of TREK-1 agonist aggravated the activation of A1-like astrocytes, while pretreatment with PDTC reversed this effect. Taken together, the findings offered the first evidence for involvement of the NF-κB pathway in the effects of TREK -1 on A1-like astrocyte activation.

However, the current work has several limitations. First, we only administered spadin to block the TREK-1 channels, but gene knockdown was not done. Although spadin may completely inactivate TREK-1 through channel blockage and internalization to exert antidepressant effects, the inhibitor is less specific than gene knockout or shRNA-mediated knockdown of TREK-1 expression. Therefore, confirmation of current results is necessary for future studies using astrocyte-specific TREK-1 gene knockdown or conditional TREK-1 knockout animal models. Second, we have concentrated on the hippocampus as the site for pathological studies. However, other regions of the brain are also dysfunctional in depression, requiring a deep investigation. Third, this study employed rat astrocyte cell line CTX-TNA2 for in vitro studies to reduce the number of experimental animals, which is an extensively used cell line in previous studies due to its similarity with primary astrocytes [45]. However, it cannot completely reproduce the features of primary astrocytes. Fourth, the potential molecular mechanism between TREK-1 and NF-κB pathway requires to be deeply explored in the future. Nevertheless, the current work has the novelty of providing information on the molecular mechanisms of TREK-1 in regulating the complex pathophysiology of MDD.

## Conclusion

**Fig. 8:**
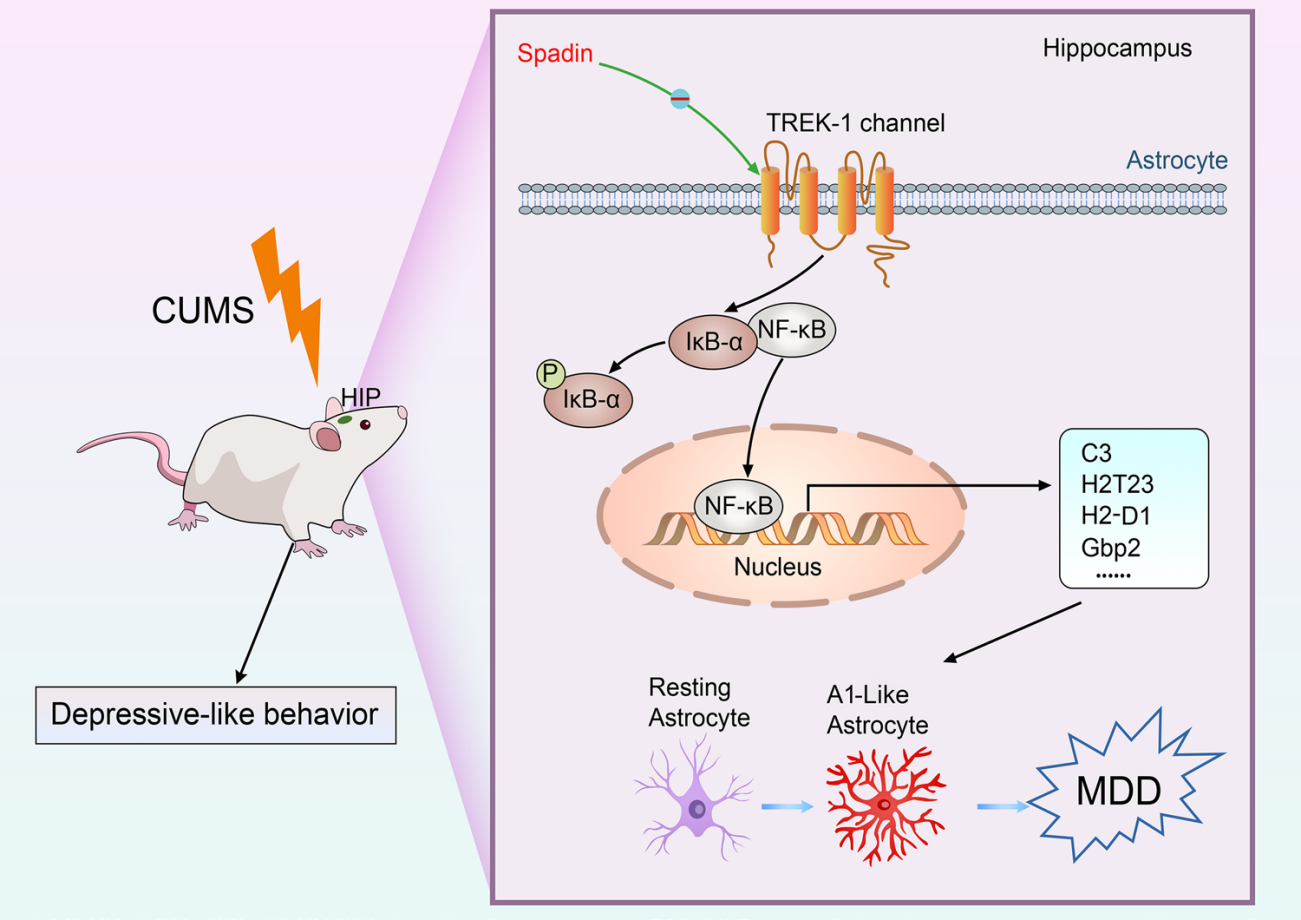
Sketch map presenting the impacts of TREK-1 on A1-like astrocyte reactivity in MDD. Blocking TREK-1 by spadin hindered A1-like astrocyte activation in the hippocampus of CUMS rats through the NF-κB signaling pathway

In summary, our study indicated that blocking TREK-1 alleviates depressive and anxiety-like behaviors of CUMS rats and hinders A1-like astrocyte activation by suppressing the NF-κB pathway in MDD via in vivo as well as in vitro studies (Fig. 8). Although additional exploration is still needed, our results provided a novel antidepressant molecular mechanism of TREK-1 action and suggested that the A1-like astrocyte may become a possible new target for treating MDD.

## Supplementary Information

Below is the link to the electronic supplementary material.

Supplementary file1 (DOCX 837 kb)

## Data Availability

The datasets generated during and/or analysed during the current study are not publicly available due to [REASON(S) WHY DATA ARE NOT PUBLIC] but are available from the corresponding author on reasonable request.
