# Supplementary material for: Blocking Two-Pore Domain Potassium Channel TREK-1 Inhibits the Activation of A1-Like Reactive Astrocyte Through the NF-κB Signaling Pathway in a Rat Model of Major Depressive Disorder
Source: Neurochem Res. 2023 Jan 20;48(6):1737–54. doi: 10.1007/s11064-023-03857-4 (PMC10119044; doi:10.1007/s11064-023-03857-4)
Supplement: Supplementary file 1 — Supplementary file1 (DOCX 837 kb) [file 11064_2023_3857_MOESM1_ESM.docx]

**
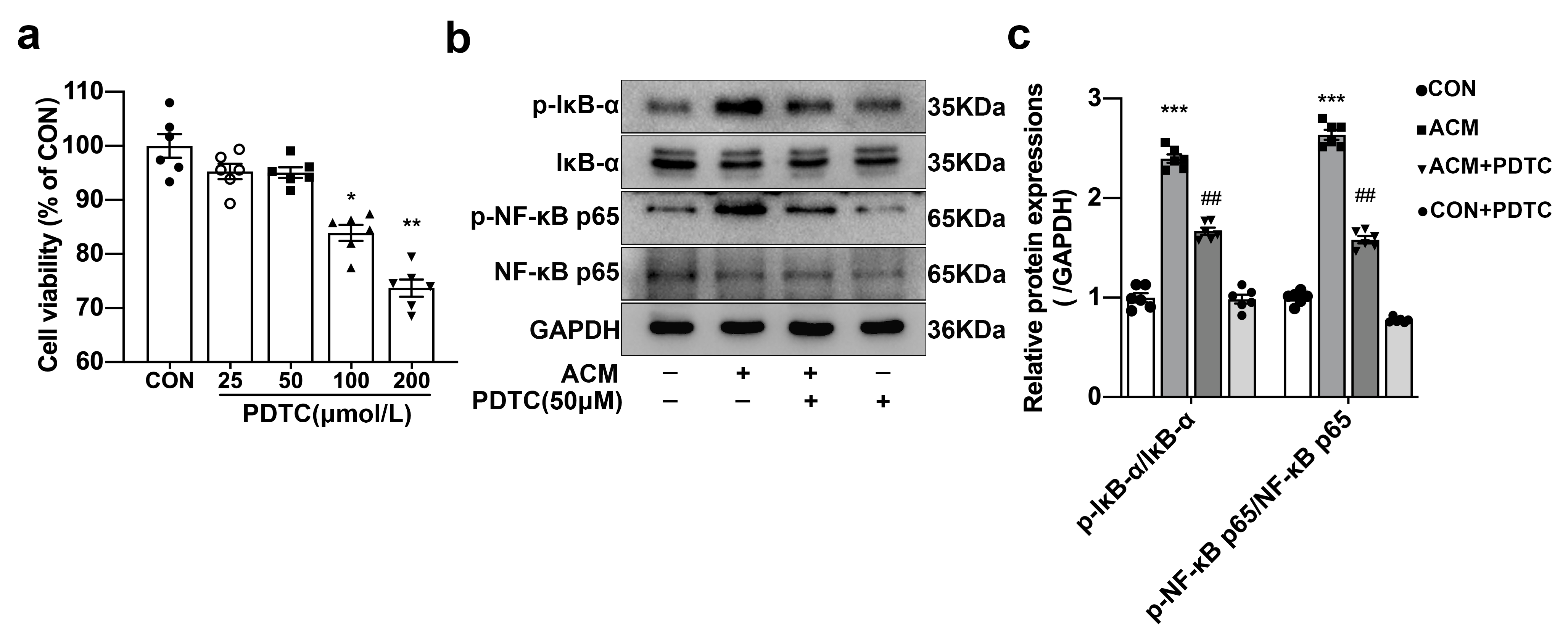
**

**Fig. S1** The effect of PDTC on astrocyte viability and the NF-κB pathway i*n vitro*. **(a)** The cell viability of astrocyte upon various dosages of PDTC pretreatment for 1 h. **(b, c)** Western blot of p-IκB-α, total IκB-α, p-NF-κB p65, and total NF-κB p65 protein levels after ACM and PDTC (50μM) treatments. Immunoreactive bands for phosphorylated protein were exposed to normalization with the unphosphorylated one. Dates are shown to be mean ± SEM. n = 6/group, **p* < 0.05, ***p* < 0.01 and ****p* < 0.001 vs. CON group; ^##^*p* < 0.01 vs. ACM group
